# Establishing Behavioural Thresholds for Dogs in Animal-Assisted Services: Expert-Derived Thresholds and Field Study Comparison

**DOI:** 10.3390/ani16071078

**Published:** 2026-04-01

**Authors:** E. Kathalijne Visser, Anna L. Jens, Peter van Honk, Mariska van Asselt, Sandra C. Haven-Pross

**Affiliations:** 1 Department of Applied Research, Aeres University of Applied Sciences Dronten, De Drieslag 4, 8251 JZ Dronten, The Netherlands; a.jens@aeres.nl (A.L.J.); p.van.honk@aeres.nl (P.v.H.); m.van.asselt@aeres.nl (M.v.A.); s.haven@aeres.nl (S.C.H.-P.); 2 Behavioural Science Institute, Radboud University, P.O. Box 9104, 6500 HE Nijmegen, The Netherlands

**Keywords:** animal-assisted services, animal welfare, dog welfare, positive welfare, expert-derived thresholds

## Abstract

Animal-Assisted Services (AASs) are increasingly used in therapeutic, educational, and supportive contexts, raising growing concern for the welfare of the dogs involved. This study assessed dogs’ affective states during AAS sessions by establishing expert-derived behavioural thresholds and comparing these with field observations. Thirteen experts in canine behaviour, health, welfare, and AAS evaluated 19 behaviours across 11 fictional scenarios using an absolute scoring approach. Expert ratings were used to derive lower and upper frequency thresholds distinguishing excellent, neutral, and unacceptable welfare for behaviours associated with positive- or negative-affective states. Field data were collected over a two-month period from 837 AAS sessions involving 63 dogs, scored by trained professionals using the same ethogram. Stress-related behaviours were generally within the expert-defined acceptable ranges, although some dogs exceeded the upper thresholds for behaviours such as low posture or sniffing. In contrast, positive-affective behaviours—including play, voluntary lying down, and broad tail wagging—were observed infrequently and often fell below the expert-defined minimum thresholds. These findings suggest that while overt stress indicators are largely managed in AAS practice, opportunities for dogs to express positive-affective states may be limited. Integrating expert-derived behavioural thresholds into welfare monitoring may support evidence-based standards and safeguard canine welfare in AASs.

## Simple Summary

Animal-Assisted Services (AASs) are widely used to support emotional, social, and therapeutic goals for people of all ages. As these services expand, it becomes increasingly important to ensure the good welfare of the dogs participating in them. This study examined how often dogs showed behaviours related to stress or positive engagement during AAS sessions and compared these observations with standards set by canine behaviour and welfare experts. We followed 63 dogs across 837 sessions and recorded 19 behaviours to evaluate their welfare. Experts established thresholds to determine when specific behaviours indicate excellent, neutral, or unacceptable welfare. Stress-related behaviours such as low posture or panting were generally within acceptable limits, although some dogs displayed these behaviours more often than experts considered ideal. Behaviours linked to positive-affective states, such as play, voluntary lying down, or broad tail wagging, were shown much less frequently than expected. These findings highlight that although dogs rarely show overt signs of poor welfare, they may have limited opportunities to express positive emotional states during AAS. Using expert-defined behavioural thresholds may help practitioners monitor dogs more consistently, identify when welfare support is needed, and ensure that dogs’ contributions to human well-being do not compromise their own quality of life.

## 1. Introduction

Animal-Assisted Services (AASs) encompass structured therapeutic, educational, and supportive interventions in which animals, most commonly dogs, are intentionally integrated to facilitate human well-being under professional supervision. A substantial body of research has demonstrated that these interventions can reduce stress and anxiety, support emotional regulation, and promote positive social interactions in human participants [1,2]. As the field expands, increasing attention has turned toward safeguarding the welfare of the participating dogs, whose emotional state is integral not only to their quality of life but also, potentially, to the stability and effectiveness of AAS sessions [3,4].

Despite this growing focus, empirical research on therapy dog welfare remains limited. Commonly used behavioural indicators such as lip licking, yawning, or panting can reflect multiple emotional states and therefore require contextual interpretation rather than simple categorization as “stress” or “comfort” [3,5]. Findings across studies are mixed: some report stable or decreasing cortisol values during AAS, while others describe subtle behavioural signs of tension or reduced engagement depending on setting, session length, and client characteristics [3,6]. This heterogeneity reflects broader methodological challenges, including variability in session structure, handler influence, and the ethograms used to classify canine behaviour [7].

At a regulatory level, the European Union formally recognises animals as sentient beings capable of experiencing both positive- and negative-affective states [8]. This recognition necessitates welfare frameworks that extend beyond preventing suffering to actively supporting positive experiences. The evolution from the “Five Freedoms” [9,10] toward the “Five Domains Model” reinforces this shift by emphasising mental state as a key component of welfare and highlighting behavioural indicators of valence and arousal [11]. Nonetheless, positive-affective behaviours—such as relaxed engagement or playful initiation—remain less consistently defined and measured than stress-related behaviours, leading to substantial variability in their operationalization [12].

Behaviour-based welfare assessment also faces challenges related to measurement accuracy. The only study directly examining human observer bias in welfare scoring found that observers expressed expectation-based bias, meaning that their evaluations shifted toward the contextual information they received, rather than showing a uniform negativity bias [13]. Moreover, video-based assessments consistently achieve higher inter-observer reliability than live observations when observers receive standardised training and clear behavioural definitions [14,15]. These insights underscore the need for clearly defined behavioural categories, structured observer training, and validation against multiple indicators.

Parallel developments in equine-assisted services demonstrate the value of defining behavioural thresholds—quantitative benchmarks that specify acceptable or unacceptable frequencies of certain behaviours. Recent consensus work in Equine-Assisted Services (EASs) has shown that such thresholds can differentiate between positive, neutral, and negative welfare states and reveal when positive-affective behaviours occur too infrequently to support optimal welfare [16]. This finding highlights a crucial point: optimal welfare requires both the absence of stress and the presence of positive, motivated engagement. Given the conceptual similarities across species, a comparable approach may be beneficial for canine welfare monitoring.

Despite widespread recognition of the importance of welfare assessment, no universally mandated minimum welfare standards exist for therapy or assistance dogs; most organisations operate through voluntary guidelines that vary considerably in implementation [17,18,19]. Key welfare practices such as rest requirements, session duration limits, and behavioural re-evaluations are applied inconsistently, and structured welfare monitoring systems are rarely used in practice [20]. Because behavioural indicators remain one of the few systematically observable sources of welfare information during AAS sessions, there is a clear need to define how often specific behaviours should occur to represent acceptable, good, or compromised welfare.

Against this backdrop, the present study aimed to (1) establish expert-derived behavioural thresholds for 19 canine behaviours typically observed during AAS sessions and (2) compare these thresholds with large-scale field data collected in professional practice. By integrating expert consensus with real-world observations, this study seeks to contribute a robust, empirically anchored framework for welfare monitoring in dogs engaged in AAS. Establishing behavioural thresholds provides practitioners with actionable benchmarks for recognising both stress-related and positive-affective behaviours, enabling earlier intervention and more consistent welfare assurance across programmes. Given that dogs’ emotional well-being is rarely measured and has never been empirically linked to intervention effectiveness, objective behavioural thresholds represent a critical step toward ensuring that their participation in AASs promotes—not compromises—their welfare.

## 2. Materials and Methods

### 2.1. Ethical Statement

This study involved only behavioural observation and expert evaluation and was conducted in accordance with Dutch legislation for non-invasive animal research. Ethical approval was granted by the Data Protection Officer at Aeres University of Applied Sciences, Dronten (protocol code AER2025-09). All human participants (experts and practitioners) provided informed consent for participation and the use of anonymized data.

### 2.2. Study Design Overview

The study consisted of two complementary components:

(1) An expert evaluation aimed at establishing welfare-related behavioural thresholds for dogs participating in Animal-Assisted Services (AASs);
(2) A comparative analysis using field data from an independent large-scale observational study of dogs working in AAS [1].

The purpose of the comparison was to evaluate whether the frequency or duration of behaviours observed in practice aligned with the expert-derived welfare thresholds. The present paper focuses on the expert thresholds’ development and their comparison with field data; detailed analyses of the field study itself are reported elsewhere [1]. A representative AAS session in this study involved 1 of 63 participating dogs engaging in an intervention (AAA, AAC, AAE, or AAT), most commonly AAT, with the session durations ranging from 25 to 48 min on average, depending on category. Across the study, 837 sessions were scored by 30 AAS handlers. During each session, a trained AAS handler conducted the intervention and, immediately afterward, systematically scored 19 predefined dog behaviours using a standardised ethogram. All observers completed approximately six hours of structured training, including instruction on behavioural definitions and a video-based assessment, and were required to achieve at least 75% accuracy before participating in the field study.

### 2.3. Expert Evaluation of Behavioural Thresholds

#### 2.3.1. Expert Recruitment

Thirteen experts in canine behaviour, health, welfare, and Animal-Assisted Services (AASs) were recruited through professional and academic networks. The eligibility criteria included a minimum of five years of professional experience in canine behaviour, health, welfare or AAS practice. The experts represented a range of domains, including clinical practice, welfare science, animal behaviour consulting, education, and AASs.

#### 2.3.2. Evaluation Procedure

Experts participated in a structured, in-person evaluation designed to establish acceptable levels of behavioural occurrence during Animal-Assisted Service (AAS) sessions. Nineteen canine behaviours commonly observed in practice were selected for assessment (Table 1). The evaluation followed an expert-based absolute scoring approach, comparable to the methodology developed within the Welfare Quality^®^ project and previously applied to horses used in therapeutic riding and equine-assisted coaching [2].

To derive upper and lower frequency thresholds for each behaviour, experts independently evaluated a standardised dataset consisting of 19 behaviours, each represented by 11 fictional scenarios. These scenarios described the increasing prevalence of frequent behavioural occurrences across sessions. Specifically, each expert received a fixed set of cases in which a dog’s behaviour was observed across ten sessions, with prevalence increasing stepwise (e.g., the behaviour was never frequently observed; frequently observed in 1 out of 10 sessions; frequently observed in 2 out of 10 sessions; frequently observed in up to 10 out of 10 sessions).

For each scenario, experts assigned a welfare score using a continuous scale from 1 to 100, where 1 represented the worst conceivable welfare state, 100 the optimal welfare state, and 50 a neutral evaluation. In accordance with the Welfare Quality^®^ framework, scores above 80 were interpreted as reflecting excellent welfare, whereas scores below 20 were considered unacceptable [2,21]. Experts were instructed to assign a score of 50 to any scenario for which they were unable to clearly classify the behavioural frequency as unacceptable, neutral, or excellent.

To ensure a consistent interpretation of behaviours, all behavioural definitions were provided in advance (ethogram; Table 1) and supported by short video clips illustrating each behaviour. The evaluation was conducted under controlled conditions to prevent mutual influence: experts were seated at separate tables and instructed not to communicate during the assessment. All scores were recorded on paper forms and subsequently entered into Microsoft Excel 365 (Microsoft Corporation, Redmond, WA, USA) for analysis.

**Table 1:** Table of behaviours assessed. Definitions and scoring categories of behaviours included in the study, with reference to the sources on which the definitions were based.

Behaviour Label	Definition	Scoring Categories	Sources
Wide, slow tail wag	A broad, slow, controlled movement of the tail. The tail swing, observed from the base of the tail, moves in the shape of a half circle (>90 degrees).	Absent: 0% of the session Occasionally: 1 to 25% of the session Frequently: more than 25% of the session	[3,4]
High posture	Breed-specific posture in which the following three behaviours are observed: elevated tail, elevated head, and ears oriented forward. These behaviours may vary in intensity but are clearly present.	Absent: 0% of the session Occasionally: 1 to 5% of the session Frequently: more than 5% of the session	[5]
Low posture	Breed-specific posture in which at least two of the following behaviours are observed: low tail, ears oriented backward, and/or flexed hind legs. At least two behaviours must be clearly visible.	Absent: 0% of the session Occasionally: 1 to 5% of the session Frequently: more than 5% of the session	[5]
Interaction with handler	The dog is in physical contact with the handler or looks at the handler while the distance between the dog’s nose and the handler’s head decreases. Alternatively, the dog follows or remains around the handler within a radius of two times the dog’s body length.	Absent: 0% of the session Occasionally: 1 to 25% of the session Frequently: more than 25% of the session	[6,7]
Interaction with client	The dog is in physical contact with the client or looks at the client while the distance between the dog’s nose and the client’s head decreases. Alternatively, the dog follows or remains around the client within a radius of two times the dog’s body length.	Absent: 0% of the session Occasionally: 1 to 25% of the session Frequently: more than 25% of the session	[5,6]
Out of sight	The dog is for an extended period (>3 min) in a location where the handler cannot observe the dog’s behaviour.	Absent: 0% of the session Occasionally: 1 to 25% of the session Frequently: more than 25% of the session	
Lying down	The dog is lying down and is not involved in the interaction or situation.	Absent: 0% of the session Occasionally: 1 to 25% of the session Frequently: more than 25% of the session	[8,9,10,11]
Panting	Increased respiration with an open mouth, where the tongue is visible inside or outside the mouth.	Absent: 0% of the session Occasionally: 1 to 5% of the session Frequently: more than 5% of the session	[5,12,13]
Avoidance/backing up	The dog moves its body or body part in the opposite direction of an offered stimulus (client, handler, object, or other stimuli).	Absent: 0 times throughout the session Occasionally: 1 to 2 times throughout the session Frequently: more than 2 times throughout the session	[11,14,15,16,17,18]
Yawning	A slow, deep inhalation visible through a widely opened mouth and jaws.	Absent: 0 times throughout the session Occasionally: 1 to 5 times throughout the session Frequently: more than 5 times throughout the session	[5,10,13,14,16,19,20]
Self-grooming	Self-care behaviour including licking, scratching, and/or nibbling parts of the dog’s own body.	Absent: 0 times throughout the session Occasionally: 1 to 5 times throughout the session Frequently: more than 5 times throughout the session	[5,22,23]
High, stiff, fast tail wag	Repeated stiff (short amplitude) and rapid side-to-side movement of the tail in a position higher than the neutral breed standard.	Absent: 0 times throughout the session Occasionally: 1 to 2 times throughout the session Frequently: more than 2 times throughout the session	[3,4,5]
Low, stiff, fast tail wag	Repeated stiff (short amplitude) and rapid side-to-side movement of the tail in a position lower than the neutral breed standard.	Absent: 0 times throughout the session Occasionally: 1 to 2 times throughout the session Frequently: more than 2 times throughout the session	[3,4,24,25,26]
Sniffing the ground	The nose moves along the ground with clear sniffing movements, not related to food.	Absent: 0 times throughout the session Occasionally: 1 to 5 times throughout the session Frequently: more than 5 times throughout the session	[12,15,24,25,26,27]
Play behaviour	Behaviour related to play, such as play bows or play vocalisations, occurring individually (with or without a toy) or in interaction with a dog or human.	Absent: 0 times throughout the session Occasionally: 1 to 5 times throughout the session Frequently: more than 5 times throughout the session	[15,26,27,28,29,30,31,32]
Lip lick	A part of the tongue briefly becomes visible and moves sharply and directly along the lips and/or nose. Excluded when the dog is anticipating food or drink or has eaten or drunk within the previous minute.	Absent: 0 times throughout the session Occasionally: 1 to 10 times throughout the session Frequently: more than 10 times throughout the session	[5,12,13,17,33]
Body shake	Active shaking of the body involving more than just the head. Movements are more abrupt and larger than trembling.	Absent: 0 times throughout the session Occasionally: 1 to 5 times throughout the session Frequently: more than 5 times throughout the session	[5,19,24,34]
Slowed movement	Slowed body movement compared to the dog’s normal pace when moving from one point to another, possibly recognisable by incomplete movements.	Absent: 0 times throughout the session Occasionally: 1 to 2 times throughout the session Frequently: more than 2 times throughout the session	[35]
Vocalisation to person	Loud or subdued barking, growling, howling, whining, or squeaking while oriented toward a person. Not related to play, food, or a given command.	Absent: 0 times throughout the session Occasionally: 1 to 5 times throughout the session Frequently: more than 5 times throughout the session	[5,12,13,15,20,36]

### 2.4. Behavioural Scoring in the Field Study

In the field study, the same 19 behaviours evaluated by experts were scored using a three-point ordinal scale: 0 = not observed/absent, 1 = occasionally observed, and 2 = frequently observed (see Table 1).

Data from each session were aggregated at the dog level by calculating the percentage of sessions in which each behaviour occurred. Data were available for 63 dogs, and the aggregated values for these dogs constituted the comparative dataset used in the present analysis.

### 2.5. Comparison of Expert Thresholds and Field Data

The behavioural scores recorded after each session in the field study were converted into percentages of the total number of sessions per dog across the two-month study period. These percentages reflected the frequency with which behaviours were classified as observed occasionally or observed frequently. Subsequently, the expert-derived behavioural thresholds were compared with the percentages of frequently observed behaviours obtained from the field data.

Experts provided threshold values for each behaviour to determine whether a given percentage of frequent occurrence should be classified as reflecting an excellent, neutral, or unacceptable welfare state. Thirteen experts participated in this desk-based evaluation.

For each behaviour, an individual expert’s data were excluded from further analysis if one or more of the following criteria were met: (1) the expert assigned a score of 50 to all 11 scenarios, indicating an inability to discriminate between welfare categories; (2) the expert’s scoring pattern lacked a monotonic (linear) trend across increasing behavioural prevalence, as determined by visual inspection; or (3) the direction of the expert’s scoring contradicted that of at least 75% of the other experts. The 75% agreement criterion was selected in line with commonly applied consensus thresholds in expert-based assessment methodologies [37].

### 2.6. Conceptual Framework

The interpretation of the expert-derived thresholds followed the Five Domains Model [11], integrating behavioural indicators of both negative (stress-related)- and positive (affiliative or relaxed)-affective experiences. The comparative analysis was designed to identify potential gaps between expert-defined welfare ideals and the behavioural patterns observed in practice, thereby providing a foundation for the future development of welfare monitoring tools and training standards in AASs. Within this conceptual framework, the incorporation of expert-derived behavioural thresholds offers a theoretically grounded and practically accessible approach to welfare monitoring.

## 3. Results

### 3.1. Expert-Derived Behavioural Thresholds

Thirteen experts with a minimum of five years of professional experience independently evaluated 19 canine behaviours across 11 fictional scenarios representing increasing proportions of sessions in which each behaviour was frequently observed. Of these experts, two specialised in canine behaviour and welfare, two in canine health and welfare, seven in canine behaviour and a form of canine-assisted services, and two in canine-assisted services only. Each scenario was rated using an absolute welfare evaluation scale ranging from 1 (very poor welfare) to 100 (excellent welfare). The mean welfare ratings exhibited clear, systematic, and monotonic patterns across scenarios. For stress-related behaviours, welfare ratings progressively declined as behavioural frequency increased, whereas for positive-affective behaviours, ratings increased with greater frequency.

Based on these consistent rating patterns, four behaviours—lying down, play behaviour, interaction with the client, and wide, slow tail wagging—were classified as positive-affective behaviours, while the remaining fifteen behaviours were classified as stress-related. Expert ratings were subsequently used to derive lower and upper frequency thresholds delineating unacceptable versus excellent welfare outcomes for each behaviour (Table 2). For stress-related behaviours, frequent occurrence was associated with low upper thresholds, indicating limited tolerance for repeated expression. For example, welfare was judged unacceptable when low posture occurred frequently in more than 21% of sessions, whereas welfare was considered excellent when this behaviour was observed frequently in fewer than 4% of sessions. Similarly, frequent lip licking was deemed unacceptable when it exceeded 45% of sessions and excellent when observed in fewer than 4% of sessions.

In contrast, behaviours classified as positive-affective displayed the opposite pattern, with higher frequencies corresponding to more favourable welfare assessments. Wide, slow tail wagging was rated as excellent when it occurred frequently in at least 83% of sessions and unacceptable when observed in fewer than 30% of sessions. Comparable threshold patterns were observed for lying down, play behaviour, and interaction with the client, all of which showed high upper thresholds and low lower thresholds, reflecting their positive association with canine welfare.

Among the stress-related behaviours, low posture had the lowest tolerance threshold (21%), followed by vocalisations to person (32%) and being out of sight (33%). For these behaviours, welfare was judged unacceptable when they were frequently observed in more than the indicated proportion of sessions. The standard deviations for these upper thresholds were relatively small (4–10%), indicating high agreement among experts regarding the maximum acceptable frequency of stress-related signals.

For positive-affective behaviours, experts defined minimum occurrence levels: play behaviour needed to be frequent in at least 14% of sessions, voluntary lying down in 13%, and interaction with the client in 26% of sessions to reflect adequate welfare. Compared with the stress-related thresholds, the standard deviations associated with these lower thresholds were larger (15–28%), reflecting greater variability among experts in determining how often positive behaviours should occur to indicate good welfare.

### 3.2. Comparison with Field Data

**Figure 1:**
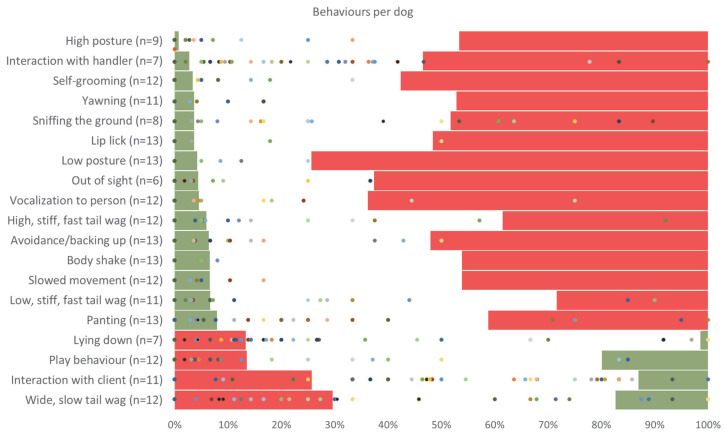
Presentation of the comparison between the current status of dogs displaying specific behaviours during field study sessions and the upper and lower thresholds established by 13 experts (where ‘n’ denotes the number of experts involved in setting the threshold for each behaviour). The percentages on the horizontal axis represent the proportions of sessions within a given time period. Each dot represents a dog. The specific numerical distribution of dogs across these welfare zones is detailed in Table 4. The behaviours are arranged according to their lower thresholds. A green–white–red ordering indicates behaviour associated with a negative-affective state, whereas a red–white–green ordering indicates behaviour linked to a positive-affective state.

The comparison between the expert-derived behavioural thresholds and the frequencies observed in the independent field dataset revealed clear and contrasting patterns between behaviours associated with negative- and positive-affective states (Figure 1). In the field study [1], each behaviour was scored as not observed, occasionally observed, or frequently observed during 837 Animal-Assisted Service (AAS) sessions involving 63 dogs. The results of the sessions are shown in Table 3. Dogs participated on average (STDEV) in 13.3 (12.3) sessions, with a minimum of 1 session and a maximum of 55 sessions over a two-month period.

For each dog, the percentage of sessions in which a behaviour was frequently observed was calculated and compared against the expert-defined lower and upper thresholds. Behaviours identified by experts as indicative of a negative-affective state (stress-related behaviours) were generally considered excellent when frequently observed in only a small proportion of sessions, as reflected by low upper thresholds (Figure 1, green-to-red ordering). In the field dataset, several of these stress-related behaviours exceeded the expert-defined maximum acceptable frequencies for a number of dogs. For example, behaviours such as interaction with the handler, sniffing the ground, vocalisations to a person, high and low stiff and fast tail wagging, and panting were frequently observed in percentages of sessions that fell within the red (unacceptable) range for multiple individuals. This indicates that, according to expert judgement, these behaviours occurred too frequently in the field to be considered compatible with acceptable welfare.

In contrast, behaviours identified by experts as indicative of a positive-affective state were considered excellent when frequently observed in a large proportion of sessions and unacceptable when rare (Figure 1, red-to-green ordering). For several of these positive-affective behaviours, including play behaviour, wide, slow tail wagging, and interaction with the client, the field data showed that many dogs fell within the unacceptable or neutral ranges rather than the excellent range. For instance, wide, slow tail wagging was frequently observed in fewer sessions than experts deemed desirable for a considerable number of dogs, placing these observations in the red or white zones of Figure 1. While Figure 1 illustrates the individual distribution of dogs relative to the thresholds, Table 4 provides a detailed numerical breakdown of the number of dogs falling within the excellent, acceptable, and unacceptable welfare zones for each behaviour.

Overall, the comparison demonstrates that some stress-related behaviours were often observed too frequently, whereas positive-affective behaviours were often observed too infrequently when evaluated against expert-derived welfare thresholds. These findings highlight a mismatch between expert expectations for optimal canine welfare during AAS sessions and the behavioural patterns observed in practice, underscoring the value of expert-informed thresholds for interpreting field-based behavioural data.

## 4. Discussion

### 4.1. Expert Consensus and Variability

The experts demonstrated a consistent pattern in their welfare evaluations across behaviours, with negative-valence behaviours judged detrimental when frequently observed, and positive-valence behaviours judged insufficient when too rare. The lower thresholds ranged from approximately 21% to 65% of sessions for stress-related behaviours, whereas for positive-affective behaviours, the minimum occurrence levels ranged from approximately 13% to 30% of sessions.

The limited tolerance for frequent stress-related behaviours such as low posture, panting, and yawning aligns with prior research identifying these behaviours as reliable indicators of discomfort or emotional strain in dogs [38,39,40]. At the same time, several commonly used stress indicators, like panting, yawning, and lip licking, may reflect multiple emotional states and therefore require careful contextualisation to avoid misclassification [14,41]. Beyond the interpretation of stress-related behaviours, experts also emphasised the importance of positive-affective behaviours, such as play, voluntary lying down, and social interaction, which were deemed necessary to indicate good welfare. This reflects the broader paradigm shift in welfare science from preventing negative states toward promoting positive experiences [42,43].

The variability among experts was greater for positive behaviours than for stress-related ones. This pattern is also reflected in the threshold data. The upper-threshold scores for stress-related behaviours clustered tightly (STDEVs of approximately 4–10%), whereas the lower-threshold scores for positive-affective behaviours showed substantially wider dispersion (STDEVs of approximately 15–28%). In other words, experts were relatively consistent in judging when stress-related behaviours occurred too often, but differed more in their views on how frequently positive behaviours such as play, voluntary lying down, or broad tail wagging should occur to indicate good welfare. This mirrors broader findings that positive welfare behaviours—such as play or relaxed engagement—are less consistently operationalised across studies than stress-related indicators [39,44]. Importantly, this variation does not undermine the collective patterns identified here; rather, it highlights the emerging and still-developing nature of positive-welfare science, in which expert perspectives can complement one another to provide a richer understanding of affective indicators.

In interpreting expert variability, it is relevant to consider that human observers are themselves subject to cognitive biases when evaluating animal behaviour. Empirical evidence on such observer biases is extremely limited, with only one study directly examining human scoring bias in animal welfare contexts [45]. That study demonstrated that observers are influenced by expectation-based bias: their welfare scores shift in the direction of the contextual information they are provided. Although such effects may contribute to individual differences in expert judgement, they do not obscure the broader consensus patterns that emerged in this study. Additionally, studies on behavioural assessment show that human observers tend to underestimate brief or subtle behaviours and to overweigh highly salient events, which can contribute to variation in expert judgement [46]. This phenomenon is often attributed to a broader ‘negativity bias,’ wherein negative signals or experiences carry greater psychological weight and are more effectively recalled than positive ones [47]. This is consistent with the broader pattern observed in animal welfare science, where the recognition of stress-related indicators is comparatively well established, while the identification and quantification of positive welfare states remains less standardised. Similar behaviours have been observed in other species, where consensus on indicators of positive-affective states develops more slowly than agreement on markers of distress [48]. As most AAS studies do not include any formal assessment of animal emotional welfare, behavioural indicators remain one of the few available and systematically measurable sources of welfare information in practice. However, behavioural indicators must be interpreted within a broader welfare context, as previous studies have shown that behavioural and physiological measures in AAS dogs often diverge. For instance, several investigations have reported weak or inconsistent associations between stress-related behaviours and cortisol concentrations [38,49,50]. Such findings underline that no single indicator provides a complete picture of affective state, reinforcing the conceptual need for multi-dimensional welfare frameworks such as the Five Domains Model. Taken together, these insights suggest that, although expert thresholds may reflect individual cognitive tendencies, they provide a robust and practically meaningful foundation for welfare evaluation, particularly for clearly defined behaviours such as those included in this study.

### 4.2. Thresholds in Relation to Field Practice

The comparison between expert thresholds and field data indicates that dogs in practice generally experience welfare conditions that are broadly aligned with expert expectations, although certain gaps were identified. Specifically, stress-related behaviours were frequently observed in a higher proportion of sessions than experts considered acceptable, whereas affiliative and relaxation behaviours were frequent in fewer sessions than experts deemed desirable. This pattern may reflect the dynamic, client-centred nature of AAS sessions, in which dogs adapt to variable environments and participant needs. Comparable patterns have been reported in observational studies of therapy dogs, where playful or relaxed behaviours occurred far less frequently than expected, despite low levels of overt stress [51]. While these findings suggest that positive-affective behaviours often fell below expert-derived thresholds, this should be interpreted with caution. A low frequency of overt behaviours, such as play or broad tail wagging, does not necessarily equate to compromised welfare. Instead, it may reflect a restricted opportunity for such expressions within the inherent structure of AAS sessions. Furthermore, positive welfare in a working context may manifest as calm affiliative engagement or focused attention, which are less overt and might not be fully captured by current ethograms. In equine-assisted services, similar results were reported: horses’ negative-affective indicators were largely absent, yet positive behaviours such as exploration or social play occurred infrequently [52]. These cross-species patterns highlight a recurring welfare issue: the operational focus on the absence of stress as a welfare goal may inadvertently overshadow opportunities for positive experiences [42,43].

An additional consideration concerns whether behavioural patterns in the field might be influenced by human documentation bias rather than actual animal welfare states. Human observers may unintentionally interpret ambiguous behaviours more negatively or positively depending on contextual framing and prior beliefs [45]. Beyond specific expectation biases, a general negativity bias [47] may further explain why practitioners are often more attuned to recording stress-related signals than more subtle positive-affective indicators. As the field dataset was generated by multiple practitioners with varying training backgrounds, expectation bias could have affected which behaviours were classified as “frequent”. However, the fact that many stress-related behaviours remained within relatively low frequency ranges suggests that practitioner scoring was generally sensitive to overt welfare indicators.

### 4.3. Implications for Animal-Assisted Service Practice

The establishment of behavioural thresholds provides clear, actionable benchmarks for evidence-based welfare monitoring within Animal-Assisted Services (AASs). For practitioners, these findings translate into operational benchmarks that can guide both individual dog assessment and programme-level welfare management. For example, the frequent observation of stress-related behaviours such as panting or low posture across more than half of sessions may indicate the need to modify session durations, environmental conditions, or handler practices. Conversely, the limited occurrence of positive behaviours such as play or voluntary resting could signal insufficient opportunities for engagement or recovery.

Recent systematic reviews demonstrate that no universally mandated welfare standards currently exist for therapy, service, or assistance dogs, with most programmes relying on voluntary and variably implemented guidelines [53,54]. Implementation gaps persist in key areas such as session duration limits, rest breaks, and periodic behavioural re-evaluations, which are inconsistently applied across organisations [55,56]. This aligns with broader evaluations of working-dog welfare programmes, which consistently note the absence of mandated frameworks for routine welfare monitoring, despite strong expert consensus on their necessity [57]. Expert consensus increasingly advocates for individualised, context-sensitive welfare assessment that emphasises both negative and positive welfare indicators rather than rigid procedural prescriptions [37,58].

Integrating empirically derived behavioural thresholds into structured welfare audits, handler training curricula, and digital monitoring tools could therefore advance standardisation and strengthen welfare oversight. Given that handlers sometimes underestimate or misinterpret dogs’ behavioural stress signals, such structured tools may also help improve welfare vigilance and reduce reliance on subjective judgement [59,60]. Moreover, applying these thresholds can support scientifically grounded welfare assurance across AAS contexts [61].

Importantly, these thresholds do not function as rigid prescriptions but as supportive guidelines that can help practitioners tailor welfare considerations to the individual dog and context. At the same time, while human outcomes in AASs are well documented, animal well-being is rarely measured, and the relationship between canine emotional state and intervention effectiveness remains unexplored. This underscores the potential for behavioural thresholds to strengthen both welfare practice and scientific understanding.

### 4.4. Methodological Considerations and Limitations

The threshold approach adopted here combines expert consensus and empirical data, providing both ecological validity and practical applicability. By grounding threshold definitions in behaviours that experts consistently recognised across scenarios, the approach offers a realistic and welfare-sensitive tool for clinical application. However, several considerations provide opportunities for future refinement. First, while the expert panel (*n* = 13) proved sufficient to establish consistent monotonic patterns and a clear consensus on stress-related indicators, the higher variability observed in positive-affective thresholds suggests that subsequent studies involving even broader panels could further enhance the precision of these benchmarks. Second, this study establishes a valuable foundation within the Dutch context. Given that AAS structures and perceptions of dog behaviour can vary internationally, extending this research through cross-cultural validation will be a constructive step toward establishing global standards for canine welfare in AAS.

Beyond these demographic considerations, the temporal structure of the evaluation also warrants attention. Experts based their ratings on frequency scenarios that reflected cumulative behavioural expression across multiple sessions, thereby capturing the repeated nature of AAS work. While using a 10-session block was necessary to ensure scientific robustness and statistical comparability in this study, we recognise the practical need for more immediate feedback. In a professional setting, these thresholds can be adapted for more dynamic monitoring; for instance, observing frequent stress-related behaviours over just 2–5 sessions could serve as an early warning signal to prompt intervention before welfare is compromised. Furthermore, the scoring system itself provides immediate feedback after every single session, as the ‘frequently observed’ category for any indicator (e.g., more than 10 lip licks or 5% of the session duration for low posture) should already trigger practitioner vigilance.

In addition to the monitoring timeframe, the analytical granularity of the scoring system presents another methodological choice. The decision to use the frequently observed category as the primary analytical unit ensured direct comparability with the field dataset but also introduced a limitation: the sometimes observed category was not analysed in depth. While this restricts the granularity of some interpretations, it preserves methodological clarity and comparability, thereby strengthening the internal validity of the findings. The selection of the 19 behaviours included in the ethogram was guided by the Five Domains Model, aiming to encompass various states of valence and arousal. However, following pilot observations, the final selection was refined to prioritise behaviours that were typically observed during AAS sessions and could be practically and reliably monitored by practitioners in real time. Consequently, the ethogram may lean toward more overt behaviours, such as play or broad tail wagging, which are highly salient during sessions. It is important to recognise that, in a professional working context, positive welfare may also manifest through subtler indicators such as calm affiliative engagement or focused attention. Expanding future monitoring tools to include these subtle indicators offers a promising opportunity to further enrich our understanding of positive welfare in professional settings. To mitigate potential anatomical bias, particularly for breeds with ‘floppy’ ears or different tail types, the ethogram defines postures as ‘breed-specific’. During observer training, reference materials and video clips were used to ensure accurate scoring across diverse breeds. For ‘low posture’, the requirement of at least two out of three indicators (low tail, backward ears, or flexed hind legs) further ensures reliability when one anatomical feature is less expressive.

Ultimately, the effectiveness of these benchmarks relies not only on their categorization but also on the consistency and accuracy of human observation. While no formal inter-rater reliability test was conducted during the evaluation session, the use of standardised behavioural definitions and video examples likely supported a shared interpretative framework among experts. Previous research shows that clear operational definitions and structured observer preparation are critical for achieving acceptable inter-observer agreement, while observer drift may occur in the absence of calibration procedures [62,63]. Tuyttens et al. [45] showed that observer judgements can shift according to contextual expectations. However, because this study used standardised ethograms and video examples, these effects were likely minimised in the expert component. In the field component, observer variation may have contributed to some discrepancies, particularly for ambiguous behaviours. Recognising such influences provides a constructive path forward for developing even more robust welfare monitoring systems.

### 4.5. Toward a Welfare Balance Model in AAS

Taken together, the findings support a welfare balance model for dogs in AAS, in which both upper limits for stress-related behaviours and lower limits for positive-affective behaviours define an optimal range of welfare expression. This dual-threshold framework aligns with the “Five Domains Model” [42] and with the recent consensus on positive animal welfare as an active state of flourishing rather than merely the absence of suffering [43].

Comparable welfare frameworks emphasise that opportunities for exploration and voluntary social engagement are essential components of a positive-affective state, yet these are rarely quantified in AAS research [64]. The present thresholds operationalize these abstract principles by providing measurable indicators that practitioners can use in daily practice. Applying this framework across animal-assisted contexts can improve welfare safeguarding and professional accountability while reinforcing the ethical foundation of AASs. Establishing similar behavioural thresholds for other species (e.g., cats or small animals) could further standardise welfare assessment across the sector.

## 5. Conclusions

The expert-derived thresholds presented here offer a quantitative and transparent basis for assessing the welfare of dogs in AAS. The comparison with field data highlights the need to balance welfare assessment by considering both negative- and positive-affective indicators. However, while these thresholds provide a valuable benchmark, the observed lower frequencies of positive behaviours should not be viewed as a definitive indicator of poor welfare. Rather, they highlight that the opportunities for overt positive-affective expression may be limited by the session’s structure. Together, these findings demonstrate that behavioural thresholds can capture meaningful welfare patterns across both expert judgement and real-world practice, providing a practical tool for identifying where welfare support is already effective and where further enhancement may be beneficial. Integrating these benchmarks into professional practice will support the development of evidence-based standards that ensure that therapy dogs’ contribution to human well-being does not compromise their own quality of life. Moreover, by operationalising positive-affective behaviours alongside stress-related indicators, this framework encourages a more complete and proactive approach to canine welfare in AASs. Future research can build on this foundation by validating thresholds across different settings, dog populations, and species, thereby strengthening the scientific basis for welfare safeguarding throughout the AAS field. Future research should investigate whether more subtle indicators of positive affect, suited to a professional working environment, might provide a more complete picture of canine well-being in AASs, in addition to validating these thresholds across different settings, dog populations, and species.

## Figures and Tables

**Table 2:** The upper (excellent) and lower (unacceptable) thresholds (mean and standard deviation, STDEV) for 19 behaviours exhibited by dogs during dog-assisted interventions, derived from an evaluation exercise in which 13 experts assessed 11 fictional sessions for each behaviour. See Table 1 for definitions.

Behavioural Label	Definition of Observed Frequency or Duration	Lower Threshold (STDEV)	Upper Threshold (STDEV)
High posture	More than 5% of a 45 min session	53% (24%)	1% (2%)
Interaction with handler	More than 25% of a 45 min session	44% (30%)	3% (7%)
Self-grooming	More than 5 times in a 45 min session	39% (17%)	3% (4%)
Yawning	More than 5 times in a 45 min session	49% (29%)	4% (4%)
Sniffing the ground	More than 5 times in a 45 min session	48% (24%)	4% (6%)
Lip lick	More than 10 times in a 45 min session	45% (28%)	4% (7%)
Low posture	More than 5% of a 45 min session	21% (12%)	4% (5%)
Out of sight	More than 25% of a 45 min session	33% (39%)	4% (8%)
Vocalisations to person	More than 5 times in a 45 min session	32% (18%)	5% (7%)
High, stiff, fast tail wag	More than 2 times in a 45 min session	56% (30%)	6% (10%)
Avoidance/backing up	More than 2 times in a 45 min session	42% (30%)	6% (10%)
Body shake	More than 5 times in a 45 min session	47% (25%)	7% (8%)
Slowed movement	More than 2 times in a 45 min session	47% (25%)	7% (8%)
Low, stiff, fast tail wag	More than 2 times in a 45 min session	65% (25%)	7% (8%)
Panting	More than 5% of a 45 min session	51% (25%)	8% (10%)
Lying down	More than 25% of a 45 min session	13% (15%)	99% (4%)
Play behaviour	More than 5 times in a 45 min session	14% (18%)	80% (20%)
Interaction with client	More than 25% of a 45 min session	26% (25%)	87% (13%)
Wide, slow tail wag	More than 25% of a 45 min session	30% (28%)	83% (12%)

**Table 3:** Percentages of sessions in which each behaviour was displayed frequently, occasionally or was absent during animal-assisted intervention sessions over a two-month period.

Behavioural Label	Frequently	Occasionally	Absent
Wide, slow tail wag	26%	58%	16%
High posture	2%	29%	69%
Low posture	1%	13%	86%
Interaction with handler	17%	75%	8%
Interaction with client	58%	41%	1%
Out of sight	2%	6%	92%
Lying down	19%	43%	38%
Avoidance/backing up	4%	25%	71%
Yawning	1%	34%	66%
Self-grooming	2%	30%	68%
Panting	10%	30%	60%
High, stiff, fast tail wag	11%	21%	67%
Low, stiff, fast tail wag	10%	12%	78%
Sniffing the ground	16%	47%	37%
Play behaviour	8%	39%	53%
Lip lick	1%	48%	51%
Body shake	0%	41%	59%
Slowed movement	1%	12%	87%
Vocalisation to person	2%	19%	79%

**Table 4:** Distribution of dogs (*n* = 63) across the excellent (green), acceptable (white), and unacceptable (red) welfare zones for each behaviour. This table provides a numerical breakdown of the field study data compared to the thresholds established by the expert panel (*n* = 13), supplementing the visual representation in Figure 1.

Behavioural Label	Excellent (Green Zone)	Acceptable (White Zone)	Unacceptable (Red Zone)
High posture (*n* = 9)	53	9	1
Interaction with handler (*n* = 7)	34	24	5
Self-grooming (*n* = 12)	54	9	0
Yawning (*n* = 11)	59	4	0
Sniffing the ground (*n* = 8)	42	14	7
Lip lick (*n* = 13)	60	2	1
Low posture (*n* = 13)	58	5	0
Out of sight (*n* = 6)	59	4	0
Vocalisations to person (*n* = 12)	56	5	2
High, stiff, fast tail wag (*n* = 12)	50	12	1
Avoidance/backing up (*n* = 13)	54	8	1
Body shake (*n* = 13)	62	1	0
Slowed movement (*n* = 12)	61	2	0
Low, stiff, fast tail wag (*n* = 11)	51	9	3
Panting (*n* = 13)	46	12	5
Lying down (*n* = 7)	3	21	39
Play behaviour (*n* = 12)	2	7	54
Interaction with client (*n* = 11)	14	34	15
Wide, slow tail wag (*n* = 12)	8	11	44

## Data Availability

The original contributions presented in this study are included in the article/Supplementary Material. Further inquiries can be directed to the corresponding author.

## Supplementary Materials

The following supporting information can be downloaded at: https://www.mdpi.com/article/10.3390/ani16071078/s1, Table S1: Raw data expert sessions.
